# Genetic markers as a predictive tool based on statistics in medical practice: ethical considerations through the analysis of the use of HLA-B^*^27 in rheumatology in France

**DOI:** 10.3389/fgene.2015.00299

**Published:** 2015-10-16

**Authors:** Hélène Colineaux, Adeline Ruyssen-Witrand, Anne Cambon-Thomsen

**Affiliations:** ^1^Department of Epidemiology, Centre Hospitalier Universitaire de ToulouseToulouse, France; ^2^Unit 1027, Epidemiology and Public Health Analyses, Inserm and University Toulouse III Paul SabatierToulouse, France; ^3^Rheumatology Center, Purpan Teaching Hospital, Centre Hospitalier Universitaire de ToulouseToulouse, France

**Keywords:** HLA-B27, rheumatology, ankylosing spondylitis, predictive genetic marker, multifactorial disease, statistical prediction, ethics, legal framework

## Abstract

**Introduction:** The use of genetic predictive markers in medical practice does not necessarily bear the same kind of medical and ethical consequences than that of genes directly involved in monogenic diseases. However, the French bioethics law framed in the same way the production and use of any genetic information. It seems therefore necessary to explore the practical and ethical context of the actual use of predictive markers in order to highlight their specific stakes. In this study, we document the uses of HLA-B^*^27, which are an interesting example of the multiple features of genetic predictive marker in general medical practice.

**Materials and Methods:** The aims of this monocentric and qualitative study were to identify concrete and ethical issues of using the HLA-B^*^27 marker and the interests and limits of the legal framework as perceived by prescribers. In this regard, a thematic and descriptive analysis of five rheumatologists' semi-structured and face-to-face interviews was performed.

**Results:** According to most of the interviewees, HLA-B^*^27 is an “overframed” test because they considered that this test is not really genetic or at least does not have the same nature as “classical genetic tests”; HLA-B^*^27 is not concerned by the ethical challenges of genetic test; the major ethics stake of this marker is not linked to its genetic nature but rather to the complexity of the probabilistic information. This study allows also showing that HLA-B^*^27, validated for a certain usage, may be used in different ways in practice.

**Discussion:** This marker and its clinical uses underline the challenges of translating both statistical concepts and unifying legal framework in clinical practice. This study allows identifying some new aspects and stakes of genetics in medicine and shows the need of additional studies about the use of predictive genetic markers, in order to provide a better basis for decisions and legal framework regarding these practices.

## Introduction

The history of HLA (*Human Leukocyte Antigen)* is related to the history of transplants, the development of which has largely relied on mastering immunological rejection. Jean Dausset discovers the HLA system in 1952 (Teillaud, 2015) and its central role in immunological reaction in 1958 (Dausset, 1958). The HLA system allows distinguishing between the biological Self and the Non-Self and is the most polymorphic multi locus system known so far. On an indicative basis, 9000 different alleles were identified in 2013 (Robinson et al., 2013). The HLA encoded proteins (antigens) carried on the cell's surface allow the immune system to recognize them as being foreign or not. Now, ≪ HLA typing ≫ is performed before an allogenic transplant to find the most compatible donor and to reduce immunological rejection. Because of its huge genetic diversity, HLA is also used for population genetics for example, in order to study past migrations of human populations (Teillaud, 2015).

It is in such a context of use that the discovery of HLA association with diseases took place. From 1969 onwards, certain ≪ HLA types ≫ were shown to be statistically and geographically associated to diseases or drug intolerances (Hors, 1989). Such an association is documented for more than 200 diseases, involving diverse mechanisms (inflammatory, neoplastic, infectious…), but all “multifactorial.” Indeed, the HLA type does not completely explain the presence or absence of the disease, it is neither sufficient, nor necessary but statistically associated. The strongest association identified concerns the HLA-B^*^27 allele and the ankylosing spondylitis (Howell, 2013). The proportion of HLA-B^*^27 varies from one population to another but about 7% of Caucasian populations are positive (Van der Linden et al., 1984; Reveille et al., 2012). Less than 1% of these populations has an ankylosing spondylitis (Van der Linden et al., 1984; Braun et al., 2005; Saraux et al., 2005) but more than 80% of these patients are HLA-B^*^27 positive (Dougados et al., 2011; Howell, 2013). Therefore, the relative risk is >50. The pathophysiological link, however, is still not entirely understood. There are several hypotheses: preferential presentation of “arthritogenic peptides,” HLA-B^*^27 misfolding or formation of homodimers which increase inflammatory response (Taurog, 2010); but the exact causes of the disease remain unknown. Other genes are implied at a lower extent, as are unknown environmental factors (Costantino and Breban, 2014).

Ankylosing spondylitis is the most important group of the spondyloarthropathies (Haute Autorité de Santé, 2008). It is an inflammatory disease that primarily affects the joints of the spine, but also the peripheral joints, enthesis (tendons, ligaments) and other organs like eyes, skin, and gastrointestinal system. Chronic inflammation causes progressive ossification of the enthesis, which leads to spinal ankylosis, and thus stiffness, deformation and physical disabilities whose severity is variable.

The first symptoms of the disease have an early onset, appearing between 15 and 30 years of age, but they are not specific (back pains). And the first characteristic radiological signs, that can confirm the diagnosis, appear later. The diagnosis is rarely mentioned at first, especially as the disease is rare. For this reason, the delay between the first symptoms and the diagnosis is long, often between 5 and 6 years (Khan, 2002). The diagnosis therefore depends on a panel of arguments, principally clinical and radiological, but also biological especially with the HLA-B^*^27 test. Indeed, even if the physiological mechanisms underlying this association still remain uncertain. HLA-B^*^27 has been rapidly used by general practitioners and rheumatologists as an element to orientate diagnostic, already before DNA tests existed, based on protein level testing.

Thus, HLA-B^*^27 has been used by rheumatologists for a long time, but it never occupied a key place in the diagnostic process, it was just an argument among many others, since the positive predictive value (1–10% in the general population) is not so important (Haute Autorité de Santé, 2013). There has been however an important change over the last years, due to the appearance of ≪ Anti-TNF α ≫ biotherapies in the treatment of the disease, which are expensive and have secondary effects. Though the diagnostic certainty does not condition the use of an anti-inflammatory treatment, it becomes mandatory to use this new therapy. It was therefore necessary to formalize the diagnostic criteria of ankylosing spondylitis, using “objective evidence,” like HLA-B^*^27, to control the therapeutic requirements.

Initially, the HLA typing technique was serology. Then, the development of molecular biology techniques in the late 80s has improved HLA typing resolution by direct analysis of the genes encoding HLA on chromosome 6. The genotyping has become the ≪ gold standard ≫ in the 1990s.

At the same time, during the development of molecular biology techniques, questions related to medical practices (when to use this information and how to interpret it) and ethical stakes (eugenics, discrimination, etc.) were raised. It generated the need for a collective and legal response to the new ethical issues raised by biomedical science (Lenoir and Sturlèse, 1991). In France, a bioethics law, which limits and frames the use of these techniques, was enacted in 1994 (Loi n° 94-654 1994)^1^ then revised in 2004 (Loi n° 2004-800 2004)^2^ and 2011 (Loi n° 2011-814 2011)^3^.

France is an international precursor in the domain of bioethics, with the creation of its National Consultative Ethics Committee in 1983 (Décret n°83-132 1983)^4^. Over 10 years of reflection have yet been necessary to produce the first bioethics laws (Mazaulat and Mattéi, 2015). In agreement with the French ethics tradition of principlism and universalism, this is overall a protective and maximalist frame, which gradually liberalized by exemptions in the name of medical and scientific interests (for research on human embryos for example, prohibited in 1994 and then gradually allowed under conditions).

Initially, the kind of genes which are explored in medical practice and therefore pose major practical and ethical challenges are those that are directly involved in monogenic diseases. Schematically, these are genes whose certain variants are directly causal of a pathology. So they can predict the onset of a disease (e.g., Huntington, 100% of risk in case of a “positive test”) or the risk of transmission (e.g., cystic fibrosis, 25% of risk for a couple of healthy carriers). Most often, the diseases at stake are rare but serious. Thus, an access to the mutation causing the family disease opens the possibility of prenatal diagnosis, thus potentially, a termination of pregnancy. There was therefore a need for the legislation to regulate the use of these techniques.

But a wider exploration of the genome strongly challenges this deterministic view of the genome (Gayon, 2007; Darrasson, 2014). Above all, the use of epidemiological methods to link genetic information and diseases produced another type of markers. The terminology used is various: “genetic risk factors,” “genetic predisposition” “mutation with incomplete penetrance” or “markers of multifactorial diseases,” etc. It is no longer a direct relationship between a gene and a disease, but complex and variable interactions between one or more genes and environmental factors, known or unknown. HLA-B^*^27 is one of these markers.

Markers of multifactorial diseases have a contrasted use in medical practice. When their association with disease is strong, they are practically used as markers of monogenic diseases (e.g., BRCA1). However, they do not only concern serious and rare diseases, but usually more common diseases such as diabetes, cancer, inflammatory diseases, etc. They are therefore not necessarily used by medical geneticists, accustomed to interpret genetic information, to consider family and reproductive perspective, and to manage psychological, familial and societal genetic risks. On the other hand, these markers do not necessarily bear the same kind of medical and societal consequences.

However, there is currently no distinction made by French law between these categories of markers. Indeed, the French bioethics law framed in the same way any genetic information produced by cytogenetic or molecular biology techniques (Décret n° 2008-321 2008)^5^.

In this context, it seems necessary to explore the practical and ethical context of the actual use of these markers and their legal implications, especially through the perceptions of practitioners involved in these issues. Indeed, documenting their use in relation with prediction would highlight the specific challenges of these markers.

Therefore, in this study, we chose to document the use of one marker of a multifactorial disease: HLA-B^*^27, because it has several unique features and a long history of clinical use in relation with ankylosing spondylitis. Indeed, as mentioned above, over years, the methods of its exploration evolved, the regulation of genetic tests and their legal definition appeared; furthermore, the use of the marker first as an element of diagnostic evolved toward becoming a mandatory element for prescribing certain treatments, due to its consideration in professional recommendations; in addition most of the time HLA-B^*^27 testing is prescribed by medical practitioners (rheumatologists and medical practitioners) who are not medical geneticists and who are not accustomed to using genetic tests. Thus, it is an interesting example of the multiple features of a predictive genetic marker in multifactorial diseases and its use in general medical practice.

## Materials and methods

This is a monocentric and qualitative study, based on interviews. The aims are to identify concrete and ethical issues of using the HLA-B^*^27 as a predictive marker in the diagnostic process and for therapeutic choice, and the interests and limits of the legal framework as perceived by prescribers.

### Interviewees

The targeted physicians in this study are the prescribing rheumatologists. Eight French rheumatologists in the region of Toulouse were contacted to participate, five of them accepted to undergo a semi-structured and face-to-face interview.

The five rheumatologists were working in the rheumatology center of Toulouse University Hospital, consisting of an outpatient department, an inpatient department and a consultations department. The activity of the center depends on 16 doctors, who have particularly developed the management of inflammatory rheumatism (polyarthritis, spondylitis) and osteoporosis (Présentation du Centre de rhumatologie, 2015).

The sample has an interesting diversity in terms of duration of exercise and work experience (See Table 1). The five rheumatologists have their principal activity in the hospitalization department, but they also have a consultation activity.

**Table 1:** **Sample description**.

**Subject**	**Status**	**Experience**
S1	University Professor—Hospital Practitioner	>25 years
S2	University Professor—Hospital Practitioner	>25 years
S3	Hospital Practitioner	10–25 years
S4	Hospital Practitioner	10–25 years
S5	University and Hospital Assistant	< 10 years

The doctors are volunteers. Information on confidentiality, terms and purpose of the study were provided and a written consent was collected before the interview.

### Grid, interviews, and analyze

In order to prepare a grid for the semi-structured interviews, we analyzed the legal, clinical and technical context of the test. A literature review was conducted with the objective of mapping the theoretical framework for the use of HLA-B^*^27. The results are detailed below.

The five interviews were performed by the same investigator and were recorded. They were held in two stages. Initially, the prescriber was asked to describe a “typical consultation” where a prescription of HLA-B^*^27 test is performed, and then a consultation of results presentation. Particular attention was paid to the difficulties reported by the physician, concerning decision, interpretation or information to the patient. In a second step, several points were detailed if they had not been spontaneously discussed, about: the genetic character of the marker; the probabilistic and predictive character of the marker; the ethical stakes and the place of legal regulation in practice.

Then we performed a thematic and descriptive analysis of the rheumatologist answers according to the axes of research: terms and limits of the use of HLA-B^*^27, ethical stakes and place of regulations.

#### a/medical framework

The currently recommended criteria for the diagnosis of ankylosing spondylitis are the Assessment of SpondyloArthritis international Society (ASAS)^6^ classification criteria (Rudwaleit et al., 2009). These criteria have a sensitivity of 83% and a specificity of 85% (see Table 2).

**Table 2:** **ASAS classification criteria for SpA**.

**ASAS Classification Criteria for Axial Spondyloarthritis (SpA) (Assessment Of Spondyloarthritis International Society 2015)**		
In patients with ≥ months back pain and age onset < 45 years		
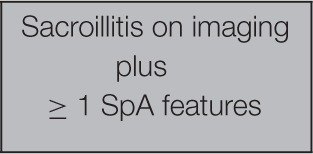	OR SpA features: Inflammatory back pain Arthritis Enthesis (heel) Uveitis Dactylitis Psoriasis Crohn's/colitis Good response to NSAIDs Family history for SpA HLA-B^*^27 Elevated CRP	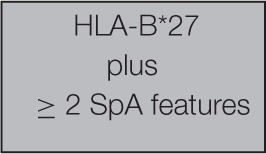

To diagnose ankylosing spondylitis, an imaging sign or HLA-B^*^27 must be present. Thus, as one of the few criteria considered as “objective,” HLA-B^*^27 has taken an important place in medical practice, through professional recommendations. Indeed it becomes a condition for biotherapy prescription if the imaging signs are not present. We note here that the “objectivity” of a criterion corresponds to the fact that it is not produced by the clinician but by a technique.

The positivity of the “HLA-B^*^27 test” is not associated with a certain kind of evolution, so it cannot theoretically be used for prognostics purposes.

#### b/legal framework of genetics in the french context

The first 1994 French bioethics Act (Loi no 94-653 1994)^7^ established the general framework principles: the principle of inviolability and non-commercialization of the human body, the necessity of a free and informed consent, and the principle of protection of the human species. It is specified that “protection of the human species” refers more specifically to the “protection of the genetic heritage.”

In the second 1994 bioethics Act (Loi n° 94-654 1994), genetics is framed more specifically. Then, the legislator decided to frame not only the intervention on the genome (gene therapy) and the use of genetics for reproductive purposes, but also all kinds of production and uses of individual genetic characteristics, based on genetic testing.

The law limits the production of and access to this information to medical and scientific research purposes. It specifically provides for obtaining written consent prior to the test, which is quite unique in the French medical practice in which consent is usually oral or even implied, especially for an exploratory and non-interventional act.

The 2004 (Loi n° 2004-800 2004) and 2011 (Loi n° 2011-814 2011) revisions of the bioethics law added items mainly related to the protection of individuals against psychological and social risk: the “right not to know,” prohibition of professional and insurance discrimination, formalization of family information, etc.

Theoretically, HLA typing is concerned by the laws regulating the use of genetic testing because it is a piece of genetic information produced by a molecular biology technique. However, it seems that this framework has been applied, in the case of HLA-B^*^27, only since the 2008 decree (*Décret n*° *2008-321* 2008), more specific about the legal definition of “genetic test,” and that its application is not uniformly considered by professionals. As an example a letter openly published by a professional underlines the perceived gap between a very strict frame of genetic testing and the practical stakes of the use of HLA-B^*^27 in rheumatology (Hatchuel, 2011).

## Results

### Actual terms of use (Table 3)

Medical doctors who prescribe HLA-B^*^27 are general practitioners, local rheumatologists and hospital rheumatologists.

**Table 3:** **Quotes about “The actual terms of uses”**.

**Subject**	**Quote**
**A FIRST LINE PRESCRIPTION**	
S1	The majority of patients come with the test already performed
S3	In 80% of cases, they already have information about B^*^27
S4	And so we have quite a few ones, coming in consultation because they were found B^*^27
S5	Sometimes [it is the primary care practitioners who prescribe them] but not always, maybe for a financial reason. Because it is not repaid if it is done in ambulatory care, so patients have to paid 80€. So, [if the practitioner thinks] that they will see the specialist at the University Hospital, it is done at the CHU. [But] when the case is simple, it's often already done
**USE BY PRIMARY CARE PRACTITIONERS**	
S1	The HLA-B^*^27 research is almost a routine examination in young people who have back pain with an inflammatory character
S1	[To orientate the patient toward a specialist] to determine whether the pain matches spondylitis knowing that HLA-B^*^27 is positive
S1	The test will be negative in 90% of cases. You have to think that there are 9 out of 10 which will not be addressed to us. […] They use it as criteria for addressing. It's worth what it's worth
S4	So all general practitioners, in particular, when they have someone who has a back pain, who is quite young, that wakes him up sometimes at night …well, they ask the B^*^27
**USE BY SECOND-LINE RHEUMATOLOGISTS**	
S1	When the diagnosis is not sure in another way, we go through the search for B^*^27
S1	The diagnosis is clinical. […] It is not a requirement. We do not need it. […] It confirms the impression of the doctor
S4	We used to ask him from time to time but it was not systematic, because, in any case, the treatment was the anti-inflammatory and, to put an anti-inflammatory, we were not bothered or controlled by the “secu”^8^. And it was also the treatment of mechanical back pain so if we were wrong it did not matter. So we asked him in hospital, for studies, to make cohorts with a relatively homogeneous population
S4	We need to be straight to the prescription of anti-TNF, although we are sure they have the disease and we don't care to know if they are or not B^*^27
S5	When the diagnosis is uncertain, as HLA-B^*^27 is now one of the axial spondylitis classification criteria, it is necessary to use it to support the diagnosis
S5	We test the B^*^27 even if we are certain of the diagnosis, because it is a component of a more comprehensive consideration
S5	We have to use it […] especially to support the use of more expensive or complex treatment such as anti-TNF
**THE MOST FREQUENTLY USE GENETIC MARKER**	
S1	In rheumatology, it is the most used
S2	Only this marker is really used
S3	HLA B^*^27 is most used

According to doctors interviewed, HLA-B^*^27 is often a first-line prescription, before addressing the patient to the hospital specialist. One of the doctors, however, reports that the refund of the test is possible only if it is done at the hospital, which can dissuade the prescription by primary care practitioners.

HLA-B^*^27 would be used for a principal reason by primary care practitioners: it is almost a routine examination, systematically prescribed in a check of back pain in young adults. The result of the test is sometimes used to orientate the patient toward a specialist, so, in a way, this is a kind of criterion for the orientation.

For the second line rheumatologists, other reasons are mentioned. HLA-B^*^27 is usually used as an argument when the clinical diagnosis is not obvious. Sometimes it is only used to confirm an obvious clinical diagnosis, or simply to complete the medical record. In this case, this criterion is sometimes collected only to provide a treatment, to be in conformity with the criteria of anti-TNFα prescription, while the diagnosis has been established in other ways.

Rheumatologists insist on the fact that the marker is not decisive in the context of this disease whose diagnosis is primarily clinical. Yet it is a very commonly prescribed test.

HLA-B^*^27 is the most frequently used genetic marker in rheumatology, according to the five doctors, far ahead of other HLA markers associated to various diseases, such as HLA-DR4 in rheumatoid arthritis, and genes of monogenic diseases (Mediterranean fever or others).

### Identified challenges with the use of the marker (Table 4)

The ethical issues reported by prescribers are not very numerous and are, for the most part, not related to the genetic nature of the marker, but rather to the fact that it is a technical tool that changes the clinical practice, and to the fact that it is a probabilistic marker.

**Table 4:** **Quotes about “The identified challenges”**.

**Subject**	**Quote**
**DIAGNOSTIC REASONING**	
S4	When you look to the criteria [in all publications, all talks], previously, there was a wide list of clinical items and so, now there are two large boxes: or I have MRI^9^, or I have HLA. It looks like that now. So all general practitioners, in particular, when they have someone who has a back pain, who is quite young, that wakes him up sometimes at night …well they ask the B^*^27. […] And so we have quite a few ones, coming in consultation because they were found B^*^27. They have backache, is that really a spondylitis? It has deviated the reasoning. That is to say, before we thought spondylitis, we made the B^*^27 to help and especially to know if we give them the TNF, now we have first the B^*^27 […] And if you cross the frequency of banal back pain, which is 80% of the population, and 8% to 10% of French people who are B^*^27, that's a lot of people who have the B^*^27 and have back pain without having a true spondylitis
**A KEY TO TREATMENT**	
S4	The problem of the disease is that it is a disease that is …Clinically they have backache, but back pain is very common. And we don't have many ways to differentiate between banal mechanical back pain and an inflammatory disease. Except the doctor's clinical judgment, there is no “objective” signs. Radios are normal for 10 years and there is no biological inflammatory syndrome. So we had two things: MRI […] and B^*^27. […] We had to find “objective criteria” to put biotherapy because it is expensive and it is not without side effects, we wanted to control prescriptions. So we look for guides and there was this B^*^27. I'm not sure that this idea is the best we have had, but as there was nothing else…
S4	We need to be straight to the prescription of anti-TNF, although we are sure they have the disease and we don't care to know if they are or not B^*^27. But we do not have enough objective arguments to get into the boxes for prescription of biotherapy and this is one easy way, since we know that 70–80% of them are HLA-B^*^27. So we said: good, there are 4 out of 5 chances he is, I would get back in the box. It's still easier to prescribe a treatment in Marketing Authorization boxes that go before a committee than to say “I depart but I'm certain”
**DISTINCTION BETWEEN THE MARKER AND THE DISEASE**	
S1	It's very difficult to explain to a patient. We know it well but to make it clear to patients, we need to start from scratch saying there is 6–8% of the population which is HLA-B^*^27 positive but I assure you there is no 6–8% of the population who has spondylitis et cetera et cetera. So we must start again on explanations…and they believe or they do not, huh…
S1	It's not so rare that patients, who have nothing or no spondylitis—they may have something else, like fibromyalgia—but were told that “you are HLA-B^*^27”, assume that they have spondylitis. And it is very difficult to reverse in terms of their understanding. […] So there is a danger to prescribe B^*^27. We come to generating anguish and pain
S2	It is quite often misunderstood. It is true that patients often make a direct association between the two: either because it is useful to them, [in a socio-professional point of view], to work stoppages or something like that; or because no one told them that they had a strong possibility of being B^*^27 without having the disease
S3	We're used, but it is not so obvious. We give them numbers and they understand. They are in demand of numbers. But to know, we should ask them at the end of the consultation what they have really understood or not
S3	It's very difficult to contest a diagnostic, what has already been told them or the conception they have formed
S5	They must understand that the marker is not the disease and the disease is not the marker, so there be no misunderstanding […], no erroneous, [and therefore no abusive prescriptions]. Generally they understand when time is taken to explain it to them, time that we have when they are hospitalized which is not necessarily the case in consultation
S5	There are patients who have been tested, which are B27 positive, who has been told “you are SpA because you are B27” […] and there, when you explain them they do not have spondylitis […], while they are built a life around it, you give a big kick in the house of cards
**USE IN ASYMPTOMATIC PATIENTS**	
S1	They sometimes ask [to test their children]. […] But they are told that being B^*^27 does not mean being sick and that the disease do not spread through B^*^27. It is explained to them and they understand that anyway we are not capable to prevent the disease, so that it does useless to create anxiety
S3	[Descendants screening] is useless, but it's a common question
S4	Luckily there are not too many [offspring tested] because we try every time to properly explain that […] it is useless, but there are some occasionally. […] In the beginning there were quite a few, now there are less
S5	I have not crossed [tested children] since a long time. I've seen a few that have been tested a long time ago, maybe 15–20 years ago when we had less insight on the B^*^27 and when people were perhaps too euphoric and optimists about this typing

One of the practitioners identified ethical issues associated with the use of HLA-B^*^27 as a “technical instrument,” i.e., instrument which, although useful, modifies the original clinical practice of the medical specialty. This tool would modify the diagnosis process and the therapeutic process in a way that can be criticized. The marker changes the diagnosis reasoning and the practice because, as it has become a routine test, the medical investigations sometimes begin from HLA-B^*^27 and not from precise clinical symptoms suggesting the disease. This is a reversal of the medical process, which searches symptoms corresponding to a marker discovered by a technique instead of using a technique to explore the subjective description of the initial symptoms. Its use as a key to treatment is also questionable. As previously noted, HLA-B^*^27 is theoretically used to maximize diagnostic certainty and minimize the risk of misusing an expensive and dangerous treatment, despite all the limits of such probabilistic marker in individual situations. In practice, its use is sometimes misappropriated and turned into a purely administrative purpose, according to the rheumatologist.

But the main difficulty reported by the physicians is the complex distinction between the marker and the disease. Indeed, the probabilistic nature of the interpretation of the presence of a marker is described as hard to explain to patients as underlined by four interviewees. Moreover, an annoying consequence of misunderstanding sometimes occurs when rheumatologists have to deny a diagnosis of ankylosing spondylitis, while the patient is positive for HLA-B^*^27.

Concerning the challenges more typically reported in relation to genetics, the only issue reported by prescribers is the fact that the test is sometimes performed predictively in asymptomatic patients, in contradiction with professional recommendations. HLA-B^*^27 cannot be used as a predictive marker in an asymptomatic subject, for two reasons mentioned by rheumatologists: the predictive value of the marker is low in this case; there is no way of prevention in the event of increased risk of spondylitis. However, the test is sometimes still performed in such situations, even if rheumatologists say that this practice is rare. Most often it is for family members of patients with spondylitis, such as their descendants, that the question to perform the test arises, while they have no symptoms and are sometimes minors.

### Legal considerations: a trivial but “overframed” test?

According to the practitioners, supervision of genetic testing in general is necessary because of psychological consequences, family and procreative consequences, and social consequences (Table 5).

**Table 5:** **Quotes about “The justifications of regulation of genetic testing”**.

**Subject**	**Quote**
**SOCIAL CONSEQUENCES**	
S1	It could not be such a factor of discrimination for employment, for a position in the society or for anything, […] unlike pathological genes, deficient gens, which could have consequences.
S1	Genetics is something that must be protected when the result might be discriminating in societal terms. […]
S5	We explain to them that it's genetic sampling, there can be slippages and the occidental law…Now, if there is a genetic sample which can be discriminatory—we will not use it for that but as it concerns his genetic inheritance—there must be a consent
S5	To avoid eugenic drifts…It was the basic idea at the very beginning
**FAMILY AND PROCREATIVE CONSEQUENCES**	
S3	There are monogenic diseases for which the involvement, the consequences are serious, for which the whole future of […] his family is upset
**INDIVIDUAL CONSEQUENCES**	
S2	I think of screening for familial cancer, there is nevertheless with huge implications. […] It changes life
S3	There are monogenic diseases for which the involvement, the consequences are serious, for which the whole future of the patient […] is upset
S5	When it is the person's genetic identity, it requires some precautions

Whereas supervision of medical genetics in itself is not questioned, its application to HLA-B^*^27 is challenging. Only one of the five doctors surveyed says that he produces a written consent when prescribing the HLA-B^*^27 test, as provided by law. According to the four other doctors, written consent is not only unrealized but also unnecessary.

They give two justifications to their posture (Table 6). First, the genetic nature of the marker is questioned as the term “genetic” is considered as applying only to hereditary diseases. Even if HLA-B^*^27 is well considered as a genetic marker from a biological point of view, its link with the disease seems to make it different from classical genetic markers. Then, the test would be not concerned by the ethical issues that justify the regulation of more “conventional” genetic testing.

**Table 6:** Quotes about “HLA-B^*^27, an “overframed” test”.

**Subject**	**Quote**
**A DIFFERENT KIND OF GENETIC MARKER**	
S1	We never considered the B^*^27 exploration as a genetic test. And it has nothing to do with a genetic test which looks for a gene abnormality because what is searched for is not an abnormality of a gene. This is a normal gene. A normal gene frequently associated with a group of diseases but it is not a defective gene. So we are not in the same spirit than in the context of genetic disease, Duchesne muscular dystrophy or other. So we do not seek an abnormal gene, we seek a normal gene
S1	It is not perceived as a genetic test, it is really perceived as if we were looking for an autoantibody.
S3	But if we carry the reasoning further, HLA-B^*^27 patients may be more “normal”. If we carry the reasoning further…Maybe being HLA-B^*^27 allows them to fight other diseases. If we reason in this way, it is something normal. This is not a genetic disease
S4	I am quite sure that, for all private practice rheumatologists and all, it is a marker of the disease as is CRP^10^. Although we know that it is a gene, […] we do not link it to a genetic problem
S4	We are told that HLA-B^*^27 is positive or negative, […] therefore we did not feel like delving into the patient's genetic inheritance
S4	This is not a sick gene
S5	This is not a genetic disease diagnosis
**DIFFERENT CONSEQUENCES**	
S1	It could not be such a factor of discrimination for employment, for a position in the society or for anything. Being B^*^27 does not mean anything, unlike pathological genes, deficient, which could have consequences.
S1	Being B^*^27 never have any impact
S1	For B^*^27, I do not consider [the consent] necessary. I do not even speak about it
S1	In addition there is no [potential] genetic counseling^11^ for B^*^27
S2	It is nevertheless something that has no consequences
S3	Here the consequences of a positive HLA-B^*^27 test…There is no direct consequence, it does not change anything. We should not dramatize

The only practitioner among those interviewed, who performs the consent does not clearly identify the ethical aspects which justify it in the case of HLA-B^*^27, except its genetic nature and the global eugenic threat posed by genetics as a whole. The law is seen as a necessary safeguard against habit and tendency to trivialize frequently prescribed tests: “I'm part of the generation, and it will gradually slide…that is to say that they are almost a routine examination for me. These tests are becoming common, it's banal and it's.…These things were extraordinary few decades ago, but for me, molecular biology is that [^*^fingers snap^*^] and the next day I have the results. […] At the beginning, it was very special and complex things, even technically, and also framed in very complex ways, and now it's trivial. […] Maybe we forget…As technically we manage to do many things, we forget that these things are some special things.” (S5).

## Discussion

This qualitative study allows to identify several practical, ethical and legal issues raised by predictive genetic markers like HLA-B^*^27 in medicine.

Regarding the use of this genetic and probabilistic marker in clinical practice, this study allows to show that markers such as HLA-B^*^27, validated for a certain usage, may be used in different ways in practice: as addressing criterion, in asymptomatic patients, or as a mandatory element for prescribing a treatment, albeit in a non-systematic way.

Like any technical instrument, it changes the practice, because it tends to replace the clinic in the diagnosis and treatment process. But this observation could involve many biological or imaging devices in medicine.

Its specificity concerns in particular the probabilistic aspect of this test and it is this aspect that seems to raise the most practical and ethical difficulties. Physicians are accustomed to using probabilistic tools and consider HLA-B^*^27 as one of them, since they spontaneously designate it as “risk factor” rather than as a “marker.” However, although it is a probabilistic test, it is used in practice ignoring these characteristics. Its admitted use seems to rely on the absence of other more reliable tools, rather than on the intrinsic characteristics of the test itself.

The statistical methods and this notion of “risk factors” have gradually invaded medicine since the mid-twentieth century (Giroux, 2008, 2010). Epistemologically, many questions about these methods and concepts are still discussed. They are obviously posed in the particular case of HLA-B^*^27. For example, the concept of “risk factor” can no longer let medical doctors think on the basis of a classical physiological causality as the quantified link between the disease and the “cause” is only statistical (Skrabanek, 1994; Berlivet, 2005). Moreover, we can no longer conceive of a strict dichotomy between normal and pathological, as this entity is placed between the two and creates continuity (Giroux, 2008).

This point can explain the communication difficulties between doctor and patient. Indeed, with the exception of practitioners who are used to interpret it, this kind of information is not intuitively and obviously understandable for the reasons mentioned above. They can also explain why this marker can be seen as both normal and pathological and this disease as both genetic and non-genetic. These difficulties, which are underlying some ethical problems, require to clarify the definition and implications of the “genetic risk factors” concept for practitioners.

Concerning the legal aspects, this study documents the idea that rheumatologists question the interest of the legal framework for the use of HLA-B^*^27, as might be deduced from the letter published in 2011 by the one of them (Hatchuel, 2011).

They do not fundamentally challenge the interest of a legal framework for the use of genetic testing, but only for the use of HLA-B^*^27 which seems to be different. Indeed, a distinction is made by rheumatologists between predictive genetic markers and the genes involved in monogenic diseases. There is a distinction in terms of ethical consequences that do not seem shared between such contexts. Genetic testing in these two domains seems to be also different in term of nature, even if this is not fully expressed. Indeed, a gene directly involved in a disease is considered by rheumatologists as pathological in itself. In the case of a predictive marker, genetic information is seen as “normal.” It is simply a “risk factor” of disease. So these entities are considered different in nature, at least instrumentally.

“Genetic exceptionalism,” i.e., considering information as sensitive because of its genetic character, is consequently questioned, because these biomarkers are considered as any other ones. This challenges the ethical and legal French framework for using genetic tools that seems not adapted to the context of multifactorial diseases.

Indeed, the laws govern any access to genetic information, without distinction of nature, utility, medical meaning, or psychological, family or social consequences. Historically, these laws seem to have been designed by and for specialists in clinical genetics. Indeed, they respond to the quite specific challenges of this specialty, as witnessed by the very specific provisions on information of related parties or content of the information to be provided prior to consent (*Loi n*° *2011-814 du 7 juillet 2011 relative à la bioéthique* 2011; *Décret n*° *2008-321 du 4 avril 2008 relatif à l'examen des caractéristiques génétiques d'une personne ou à son identification par empreintes génétiques à des fins médicales* 2008). They may therefore appear inappropriate in the context of other medical specialties such as rheumatology, which is not concerned, in most cases, by certain issues of clinical genetics such as the control of the transmission of family diseases for example.

However, one can wonder whether the attitude of practitioners who consider that ethical issues, that justify the legal rules, do not effectively concern this marker is justified or if it is just a historical or practical bias of perception by the physicians.

First, regarding the individual issues that may occur, including psychological issues, it seems indeed that they are not directly linked to HLA-B^*^27 test, but rather to the diagnosis of ankylosing spondylitis. In this sense, the ethical challenge are not different from those that emerge in any medical situation of diagnosis or identification of risk factors. If genetic exceptionalism is rightly refused, there is no reason to protect more specifically this test rather than another one simply because it is genetic.

Regarding the procreative and family aspects, more specific of genetics, the conclusion is less obvious. Theoretically, HLA-B^*^27 is not concerned by these aspects, as information and testing of related parties is not recommended. Indeed, according to rheumatologists, this information in the absence of clinical symptoms, is devoid of meaning and unnecessary since it could not justify preventive treatment or measures of transmission control (antenatal or preimplantation diagnosis). However, this seems to be a point of common concern for patients; and rheumatologists; although they are not worried, they acknowledge the fact that asymptomatic descendants are sometimes tested. It would be informative to determine the actual frequency of this practice, and the actual understanding of this aspect by the patients themselves. Even if such a strict control comparable to that for tests that have most obvious family consequences is probably not justified, it seems important to encourage a deeper reflection on these aspects among rheumatologists, who are accustomed to a practice focused exclusively on the patient himself. The provision of information about these procreative and family issues in the diagnosis process of the disease should be part of the professional recommendations.

Finally, concerning collective aspects, the conclusion is not simple either. Apart from using the slippery slope argument, the question of eugenics does not arise here: on the current state of knowledge, no selection of embryos or fetuses could logically be justified by the presence of HLA-B^*^27. However, about the risks of discrimination, we can challenge the idea, defended by the majority of rheumatologists, that the marker cannot create situations of discrimination because it has no individual meaning. Indeed, from a probabilistic point of view, a higher relative risk than the general population can always raise the possibility of selection, by insurance in particular, although this risk has no impact on the medical plan. On the other hand, it is not necessarily the role of the clinician, whose concerns are and must be the patient's individual situation, to prevent such risks. Besides, the question of why the genetic characteristics must be more protected against the risk of discrimination than any other medical characteristics and history is far from being resolved. Having a parent with ankylosing spondylitis is, for example, as informative for insurance as being HLA-B^*^27, these information are however not protected in the same way by French law.

This study is limited by the small size of the sample of professionals interviewed and would require to be extended to a larger population of rheumatologists, in order to confirm and quantify these results, as well as to other prescribers and to patients.

It would also be interesting to describe the use of other predictive genetic markers and to compare them to the HLA-B^*^27 case. However, it is difficult to find such a marker as available as HLA-B^*^27, because the markers of multifactorial diseases have either a much stronger association that assimilate them to monogenic diseases (e.g., BRCA1); or a much lower association and then they are used only in very rare cases or within research protocols (TCF7L2 in diabetes, for example). But these markers are also those which are analyzed by companies offering direct to consumer genetic testing, so it is urgent and important to analyze their concrete issues. Thus, this case is an interesting example of a predictive marker use that started long ago before the legal framework for genetics was established and that is used outside the medical genetics profession. The question remains whether it is an exception or could be considered as a model for all markers of multifactorial diseases.

In any case this marker and its clinical uses underline the challenges of translating both statistical concepts and unifying legal frameworks in clinical practice.

### Conflict of interest statement

The authors declare that the research was conducted in the absence of any commercial or financial relationships that could be construed as a potential conflict of interest.

## Footnotes

^1^Loi n° 94-654 du 29 juillet 1994 Relative au don et à l'utilisation des éléments et produits du corps humain, à l'assistance médicale à la procréation et au diagnostic prénatal.1994. 94–654.

^2^Loi n° 2004-800 du 6 août 2004 relative à la bioéthique. 2004. 2004–2800.

^3^Loi n° 2011-814 du 7 juillet 2011 relative à la bioéthique. 2011. 2011–2814.

^4^Décret n°83-132 du 23 février 1983 portant création d'un Comité consultatif national d'éthique pour les sciences de la vie et de la santé. 1983. 83–132.

^5^Décret n° 2008-321 du 4 avril 2008 relatif à l'examen des caractéristiques génétiques d'une personne ou à son identification par empreintes génétiques à des fins médicales. 2008. 2008–2321.

^6^Assessment of SpondyloArthritis International Society. [Internet] ≪ ASAS Slide Collection ≫. [Last access january, 2015].

^7^Loi no 94-653 du 29 juillet 1994 relative au respect du corps humain. 1994. 94–653.

^8^Social security.

^9^Magnetic Resonance Imaging.

^10^C-reactive Protein, marker of inflammation.

^11^Corresponds, in France, in a specialist consultation for calculating the risk of transmission to offspring and providing information on the different possibilities of reproductive control of transmission.
